# Exploring an immune cells-related molecule in STEMI by bioinformatics analysis

**DOI:** 10.1186/s12920-023-01579-8

**Published:** 2023-06-30

**Authors:** Min Zhang, Jiaxing Li, Cuncun Hua, Jiayin Niu, Pengfei Liu, Guangzhen Zhong

**Affiliations:** 1 Department of Research Ward, Beijing Chao-Yang Hospital, Capital Medical University, Beijing, China; 2 Department of Urology, Beijing Chao-Yang Hospital, Capital Medical University, Beijing, China; 3 Heart Center, Beijing Chao-Yang Hospital, Capital Medical University, Beijing, China

**Keywords:** STEMI, Biomarkers, Immune infiltration, Machine learning

## Abstract

**Background:**

ST-elevated myocardial infarction (STEMI) is the leading cause of mortality worldwide. The mortality rate of heart attacks has decreased due to various preventive factors and the development of early diagnostic resuscitation measures, but the long-term prognosis remains poor. The present study aimed to identify novel serum biomarkers in STEMI patients and explored a possible new mechanism of STEMI from an immune molecular angle with bioinformatics analysis.

**Methods:**

Gene expression profiles were obtained from Gene Expression Omnibus (GEO) database. Differential gene analysis, machine learning algorithms, gene set enrichment analysis, and immune cell infiltration analysis were conducted using R software.

**Results:**

We identified 146 DEGs (differentially expressed genes) in the integrated dataset between the STEMI and CAD (coronary artery disease) groups. Immune infiltration analysis indicated that eleven cell types were differentially infiltrated. Through correlation analysis, we further screened 25 DEGs that showed a high correlation with monocytes and neutrophils. Afterwards, five genes consistently selected by all three machine learning algorithms were considered candidate genes. Finally, we identified a hub gene (ADM) as a biomarker of STEMI. AUC curves showed that ADM had more than 80% high accuracy in all datasets.

**Conclusions:**

In this study, we explored a potentially new mechanism of STEMI from an immune molecular perspective, which might provide insights into the pathogenesis of STEMI. ADM positively correlated with monocytes and neutrophils, suggesting its potential role in the immune response during STEMI. Additionally, we validated the diagnostic performance of ADM in two external datasets, which could help to develop new diagnostic tools or therapeutic strategies.

**Supplementary Information:**

The online version contains supplementary material available at 10.1186/s12920-023-01579-8.

## Introduction

ST-elevated myocardial infarction (STEMI), the most acute manifestation of coronary artery disease (CAD), is typically caused by atherosclerotic plaque rupture and subsequent occlusive coronary thrombus formation [1, 2]. Over the past decade, the mortality rate of heart attacks has decreased due to various preventive factors and the development of early diagnostic resuscitation measures, but the long-term prognosis remains poor [3]. Thus, it is imperative to investigate the pathogenesis of heart attacks and develop more effective prevention and treatment strategies.

The necrotic myocardium in the infarct area triggers an inflammatory response immediately after acute myocardial infarction. Moreover, the inflammatory response is involved in the pathological process of post-infarction heart failure. The inflammatory response promotes morphological and functional recovery in the early stage after acute myocardial infarction and largely avoids serious complications such as myocardial rupture and malignant arrhythmia. However, the overactivation of inflammatory response could lead to myocardial infarction area enlargement and aggravated ventricular remodelling, which is related to complications such as heart failure, ventricular enlargement, and cardiac insufficiency after myocardial infarction. Therefore, it is necessary to explore the role of the inflammatory response during myocardial infarction.

With the development of new technologies such as gene microarray and next-generation sequencing, great progress has been made in the identification and validation of new diagnostic and therapeutic biomarkers. The integration and analysis of multiple datasets might provide different insights. In this study, we identified a candidate diagnostic biomarker related to immune cell infiltration based on three machine learning algorithms in integrated metadata, and then validated the diagnostic performance of ADM in two external datasets, which could help us understand the molecular mechanism and biological functions of MI.

## Materials and methods

### Data collection

Acute myocardial infarction (AMI) gene expression data were collected from the Gene Expression Omnibus (GEO) database (https://www.ncbi.nlm.nih.gov/geo/). The GSE59867, GSE60993, GSE61144, and GSE62646 datasets were downloaded. GSE59867 and GSE62646 datasets were derived from the same platform (GPL6244 platform of Affymetrix Human Gene 1.0 ST Array), so we merged the two datasets as a metadata cohort. GSE60993 (derived from the GPL6884 platform) and GSE61144 datasets (derived from the GPL6106 platform) were used as the external validation. The details of all datasets are listed in Table 1.

**Table 1:** Basic information of datasets

Datasets	Platforms	Cell Type	Control Group	STEMI Group	Applications	References (PMID)
GSE59867	GPL6244	peripheral blood	46	111	Discovery	25,984,239 [24]
GSE60993	GPL6884	peripheral blood	7	7	Validation	26,025,919 [25]
GSE61144	GPL6106	peripheral blood	10	7	Validation	26,025,919 [25]
GSE62646	GPL6244	peripheral blood	14	28	Discovery	23,185,530 [26]

### Differentially expressed genes screening and gene enrichment analysis

We applied the “limma” package to correct the background and normalize each dataset. Batch effects between metadata cohort were eliminated using the “removeBatchEffect” function of the “limma” package. Differentially expressed genes (DEGs) betweenSTEMIand control samples were identified using the “limma” package with a threshold set as |log_2_ (Fold Change [FC])| > 0.5 and a false discovery rate (FDR) < 0.05. Then, based on the DEGs, we explored Gene ontology (GO) and the Kyoto Encyclopedia of Genes and Genomes (KEGG) analysis using the “clusterProfiler” package [4].

### Evaluation of immune cell infiltration

Immune cell infiltration levels were quantified with the CIBERSORT algorithm using the “CIBERSORT” package (http://cibersort.stanford.edu/). The correlation analysis of infiltrating immune cells was visualized by the “corrplot” package and the “ggplot2” package.

### Screening of hub diagnostic biomarkers based on machine learning algorithms

Three machine learning algorithms, the least absolute shrinkage and selection operator (LASSO) [5], the support vector machine recursive feature elimination (SVM-RFE) [6] and random forests (RFs) [7] were utilized to screen for hub diagnostic biomarkers. LASSO is an acknowledged method that performs well in handling high-dimensional data. In this study, we implemented the LASSO model using the ‘glmnet’ package (https://cran.r-project.org/web/packages/glmnet/index.html), and selected the optimal lambda value through cross-validation. SVM-RFE is capable of selecting the most critical features, even in datasets with a large number of features, without sacrificing model accuracy. SVM-RFE was implemented using the “rfe” function of the “caret” package, and we used 10-fold cross-validation to search for the optimal number of features. The random forests model unites the advantages of multiple decision trees to form a composite model that is more reliable, less susceptible to overfitting, and capable of processing both categorical and continuous data. Random forests were fitted using the ‘randomForest’ package. Subsequently, we obtained the hub genes by taking the intersection of the genes derived from the three machine learning models.

### Gene set enrichment analysis

The “gseKEGG” function of the R package “ClusterProfiler” was conducted to perform GSEA. Significantly enriched KEGG pathways associated with hub genes were identified [4].

### Statistical analysis

All statistical analyses were performed using R software (version 4.2.1; Rstudio, Boston, MA). Comparisons of two groups of continuous variables were performed by Student’s t-test or Mann-Whitney U test. For multiple comparisons, one-way analysis of variance (ANOVA) and Kruskal-Wallis tests were used for parametric and non-parametric data, respectively. Receiver operating characteristic (ROC) curves and the area under the ROC curve (AUC) were used to evaluate the diagnostic performances of biomarkers. Correlations were analyzed using Spearman correlation. P < 0.05 was considered statistical significance.

## Results

### Screening and functional enrichment analyses of DEGs

**Fig. 1:**
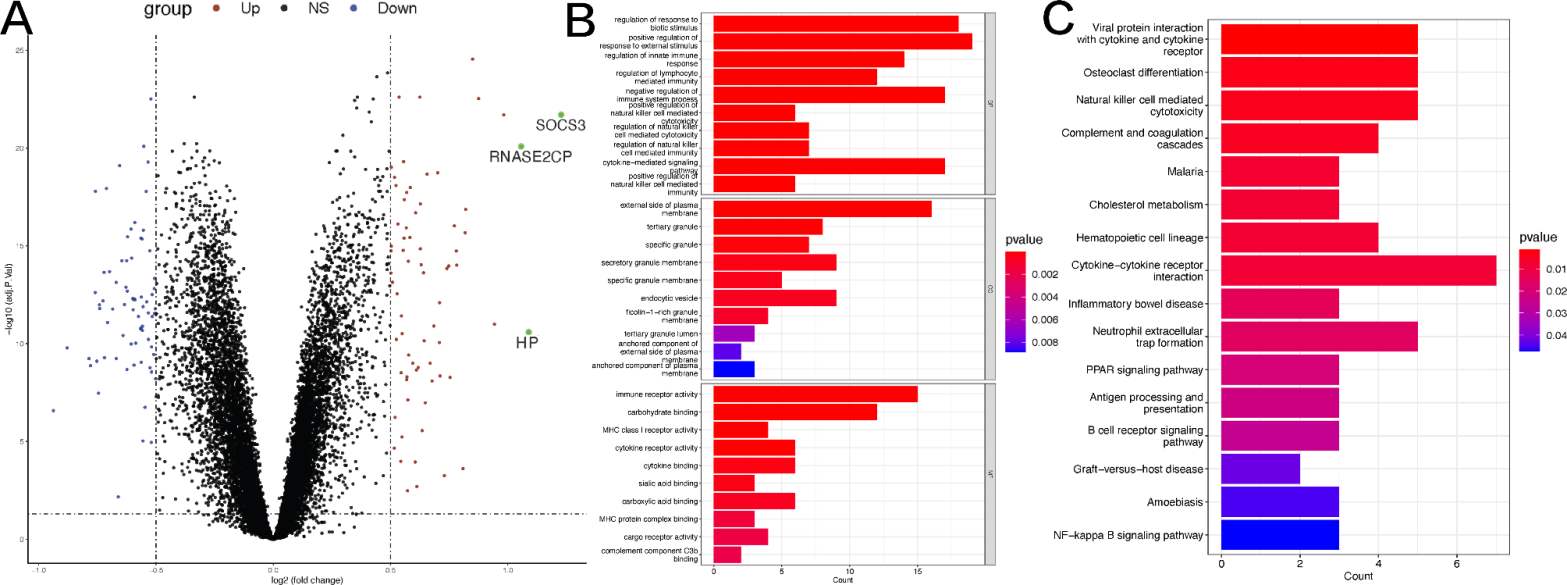
Screening and functional enrichment analysis of DEGs. (**A**) Volcano plot of DEGs. Red and blue dots represented upregulated and downregulated genes, respectively. (**B**) GO analyses. (**C**) KEGG analyses.

Differential expression analysis was conducted between 60 control samples (stable coronary artery disease) and 139 STEMI samples in the metadata cohort (GSE59867 and GSE62646 datasets). The metadata cohort before and after the batch correction is shown in Figure S1. Based on the filtering criteria, a total of 146 DEGs were obtained, including 71 upregulated genes and 75 downregulated genes (Fig. 1A). Subsequently, we performed functional enrichment analyses further to elucidate the biological functions and characteristics of DEGs. Figure 1B-C displayed the GO and KEGG analysis results, respectively. The GO annotations of DEGs consisted of several immune processes, including positive regulation of response to external stimulus, regulation of innate immune response, regulation of lymphocyte mediated immunity, regulation of natural killer cell mediated immunity, immune receptor activity, MHC class I receptor activity, and so on. KEGG analysis of DEGs also related with immune cell-related signaling pathway, such as Natural killer cell mediated cytotoxicity, Antigen processing and presentation, B cell receptor signaling pathway, NF-kappa B signaling pathway.

### The landscape of immune cell infiltration in STEMI

**Fig. 2:**
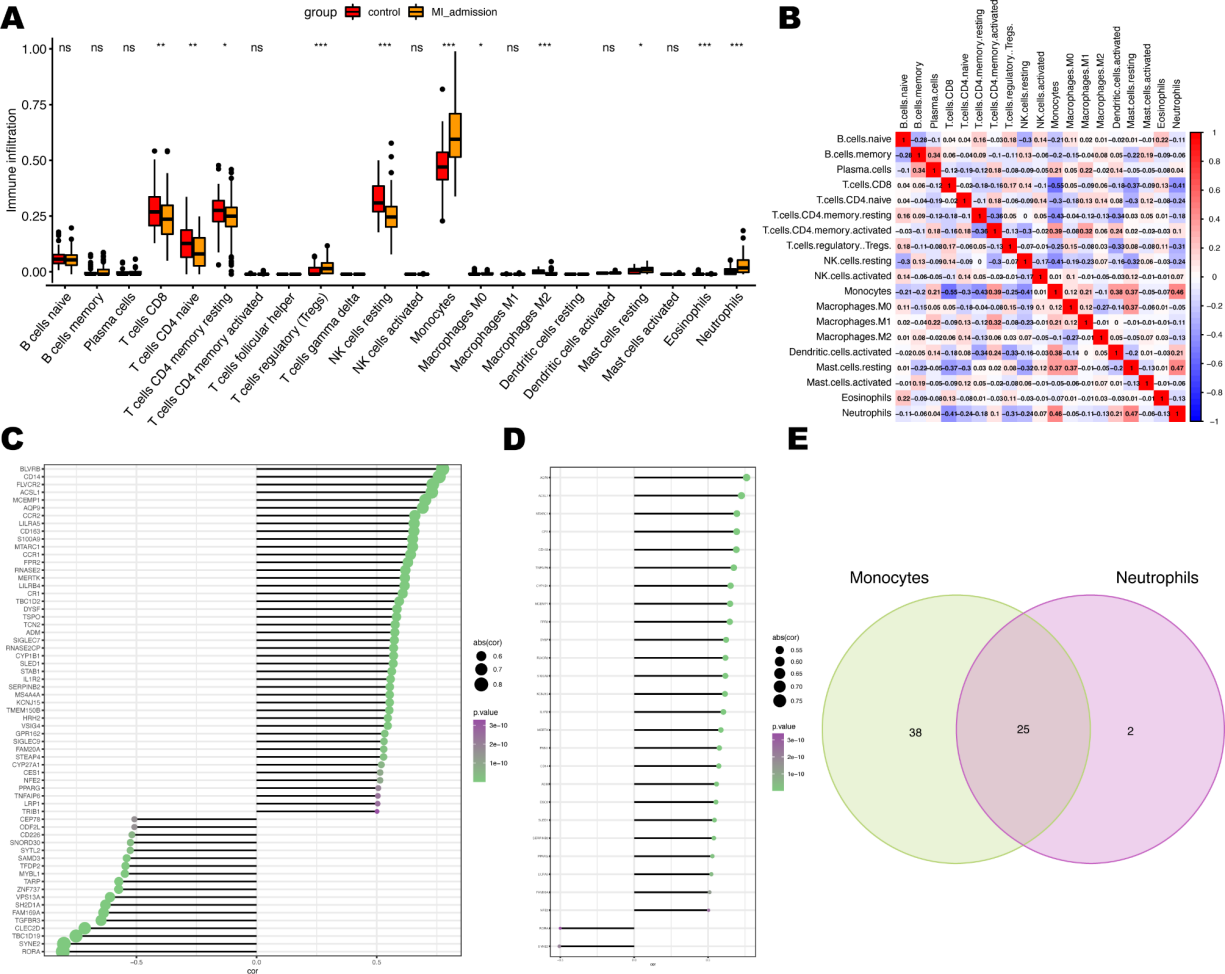
The landscape of immune cell infiltration in STEMI. (**A**) The proportion of immune cell infiltrated in STEMI and control samples. (**B**) Correlations among immune cells. (**C**) Correlations between Monocytes and DEGs (abs(cor) > 0.5 & P < 0.05). (**D**) Correlations between Neutrophils and DEGs (abs(cor) > 0.5 & P < 0.05). (**E**) Intersected genes between C and D. *P < 0.05; **P < 0.01; ***P < 0.001; ns, not significant

The immune response is important in many cardiac pathophysiological processes, including acute myocardial infarction [8]. Thus, we used the CIBERSORT algorithm to quantify the proportion of immune cell infiltration between STEMI and control samples to explore the immune features. The results indicated that infiltration abundance of T cells CD8, T cells CD4 naïve, T cells CD4 memory resting, and NK cells resting in STEMI samples were significantly lower compared to control samples, while STEMI samples had a higher infiltration proportion of T cells regulatory (Tregs), Monocytes, Neutrophils in contrast to controls (Fig. 2A). Next, we conducted the correlation analysis between infiltrating immune cells in STEMI samples (Fig. 2B). Neutrophils correlated positively with Monocytes (0.47) and Mast cell resting (0.47), while negatively with T cells CD8 (-0.41) and Tregs (-0.31). Monocytes were positively related to Neutrophils (0.46), T cells CD4 memory activated(0.39), Dendritic cells activated (0.38) and Mast cells resting (0.37), while they were negatively related to T cells CD8 (-0.55), T cells CD4 memory resting (-0.43) and NK cells resting (-0.41). Tregs cells are negatively correlated with most cell types, including Monocytes (-0.25), Dendritic cells activated (-0.33) and Neutrophils (-0.31).

### Screening of immune cell-associated differential genes

During the initial hours of acute myocardial infarction, a large number of neutrophils infiltrate ischemic myocardial tissue. Subsequently, macrophages derived from monocytes are recruited to the site of infarcted myocardium and engulf cell debris and apoptotic neutrophils. Therefore, we conducted a correlation study between monocytes and neutrophils - both of which had a high infiltration proportion in STEMI samples - and the 146 DEGs identified in STEMI samples. Differential genes with an absolute correlation coefficient greater than 0.5 and a P value less than 0.05 were selected. Out of the 63 DEGs related to monocytes, there were 45 positively correlated genes and 18 negatively correlated genes (Fig. 2C). There were 27 differential genes related to Neutrophils, with 25 positive correlations and 2 negative correlations (Fig. 2D). After taking the intersection, we obtained 25 differential genes related to immune cells (Fig. 2E).

### Hub genes identification based on three machine learning algorithms

**Fig. 3:**
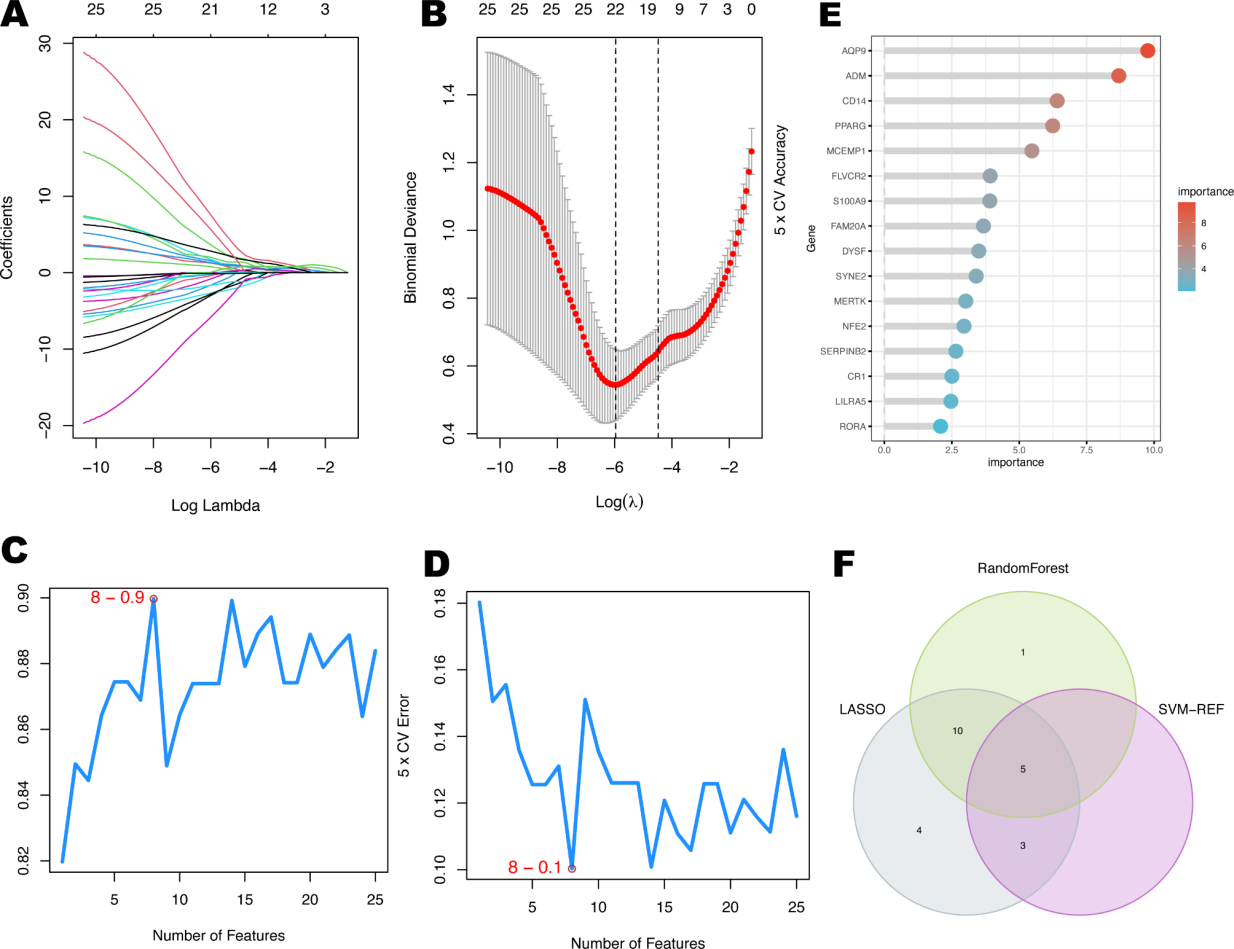
Hub genes identification based on three machine learning algorithms. (**A**, **B**) LASSO logistic regression model was applied to screen hub genes, and partial likelihood deviance with 10-fold cross-validation was utilized to calculate the best lambda. The accuracy (**C**) and the error (**D**) of the feature selection in the SVM-RFE algorithm for hub gene selection. (**E**) Random forest algorithm showed the top 16 genes with an importance greater than 2. (**F**) Venn diagram of hub genes screened by three machine learning algorithms.

Following that, we employed three machine learning algorithms to narrow down the candidate hub genes based on the 25 immune-related differential genes obtained from the previous step. According to LASSO logistic regression algorithm, we received 22 candidate genes based on the optimal value of lambda (the optimal lambda value was 0.00257) (Fig. 3A, B). Next, nine signature genes were uncovered by the SVM-RFE analysis (Fig. 3C, D). Using the RF model, we identified 16 genes with importance greater than 2, as shown in Fig. 3E. Ultimately, our findings resulted in the identification of five hub genes that were consistently identified by all three machine learning algorithms: ADM, MERTK, PPARG, RORA, and SYNE2 (Fig. 3F).

### Expression and diagnostic efficacy of hub genes

**Fig. 4:**
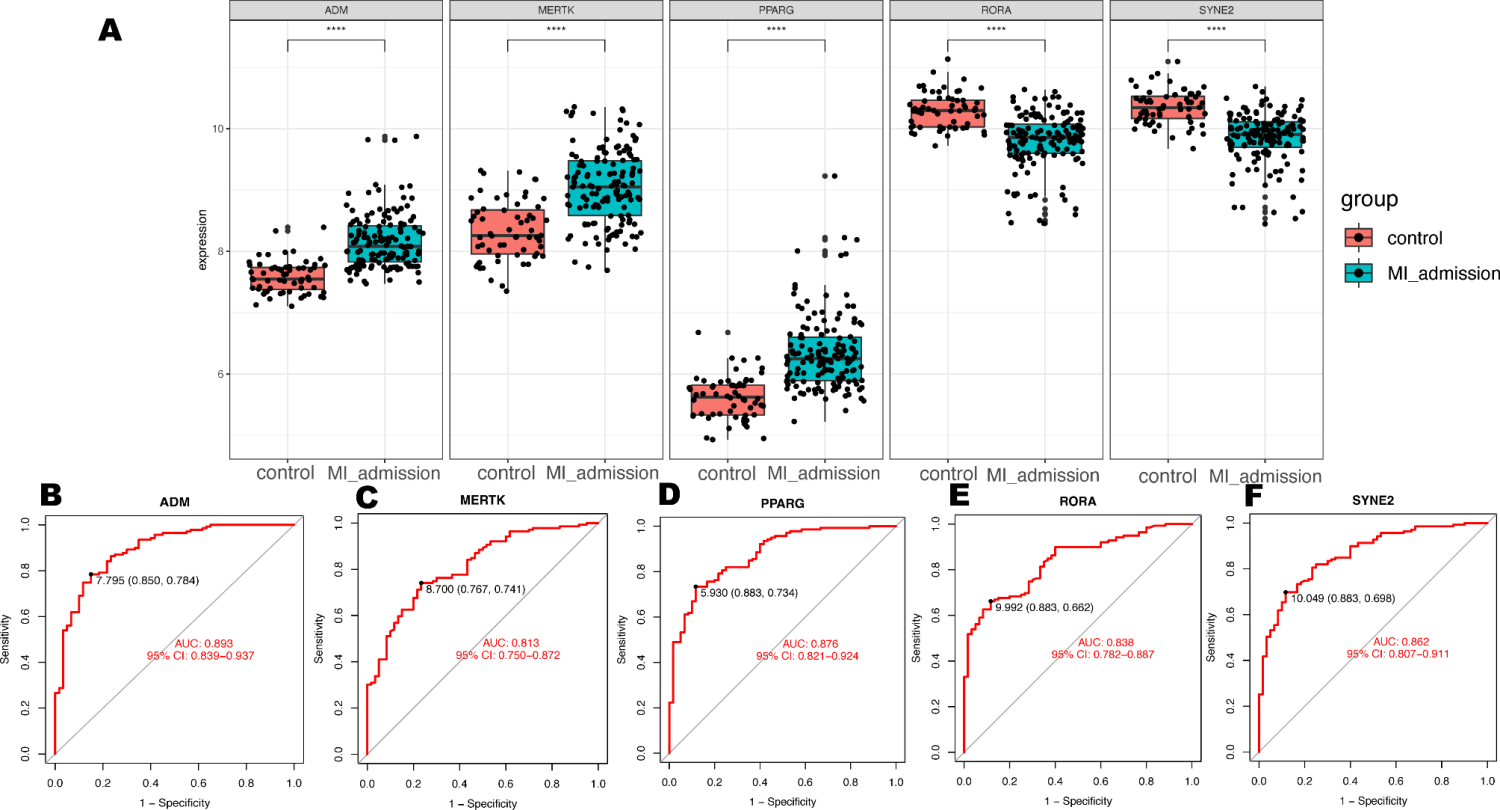
Expression and diagnostic efficacy of hub genes. (**A**) Expression of hub genes in metadata cohort. (**B**-**F**) Diagnostic efficacy of hub genes in the prediction of STEMI in metadata cohort. ****P < 0.0001

Initially, we explored the expression levels of five pivotal genes in the metadata cohort (Fig. 4A). The STEMI group showed high expression levels of ADM, MERTK, and PPARG, while RORA and SYNE2 were prominently expressed in the control group. It’s interesting that the abundance of monocytes and neutrophils has a positive relationship with ADM, MERTK, and PPARG, but a negative relationship with RORA and SYNE2 (Fig. 2C, D). The AUCs for all five genes were greater than 0.8 (Fig. 4B-F). The specific cutoff values, sensitivity, and specificity values are shown in Supplementary Table 1. Moreover, the combined ROC curve for five genes reached an AUC value of 0.941 (95% CI: 0.903–0.974) (Figure S2).

### Validation of hub genes

**Fig. 5:**
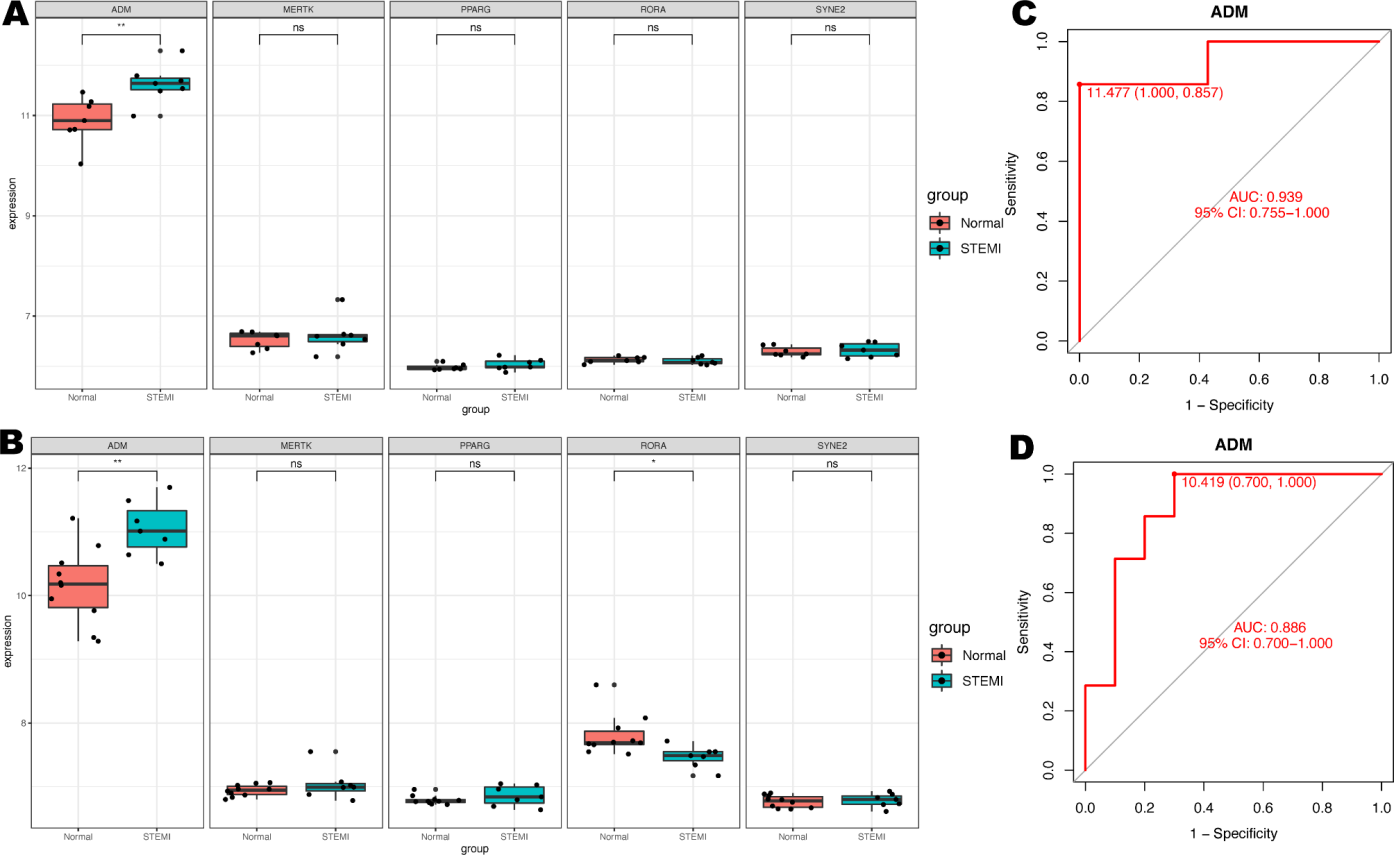
Validation of candidate diagnostic markers. (**A**) Expression of hub genes in GSE60993 cohort. (**B**) Expression of hub genes in GSE61144 cohort. (**C**) ROC curves of ADM in the GSE60993 cohort. (**D**) ROC curves of ADM in the GSE61144 cohort. *P < 0.05; **P < 0.01; ns, not significant

We applied two external datasets to validate the potential diagnostic markers. The expression levels of ADM in STEMI samples were found to be significantly elevated compared to the control samples in both the GSE60993 (P < 0.01) (Fig. 5A) and GSE61144 (P < 0.01) (Fig. 5B) cohorts. After that, we analyzed the diagnostic power of ADM. The AUC of ADM was 0.939 in the GSE60993 cohort (Fig. 5C) and 0.886 in the GSE61144 cohort (Fig. 5D), revealing ADM’s robust diagnostic performance. Additionally, we evaluated the ADM levels and its diagnostic effectiveness in the GSE59867 and GSE62646 datasets, which formed the metadata cohort. Our results showed that ADM demonstrated outstanding diagnostic accuracy (Figure S3).

### Signaling pathways associated with the candidate diagnostic markers

**Fig. 6:**
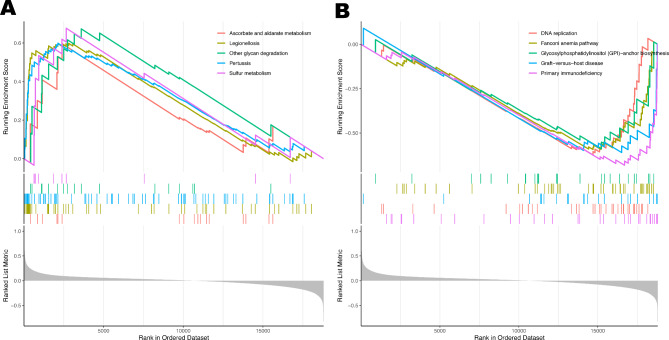
Gene set enrichment analysis in metadata cohort. (**A**) Significantly enriched pathways in high expressions of ADM. (**B**) Significantly enriched pathways in low expressions of ADM.

GSEA was conducted to seek out signaling pathways linked with the candidate diagnostic markers in the STEMI group. ADM was positively correlated with Sulfur metabolism, Other glycan degradation, and so on (Fig. 6A), while ADM showed a negative association with immune-related pathways such as Primary immunodeficiency and Graft-versus-host disease (Fig. 6B). The GSEA results for the remaining four pivotal genes are presented in Figure S4.

### Expression changes during myocardial infarction recovery

**Fig. 7:**
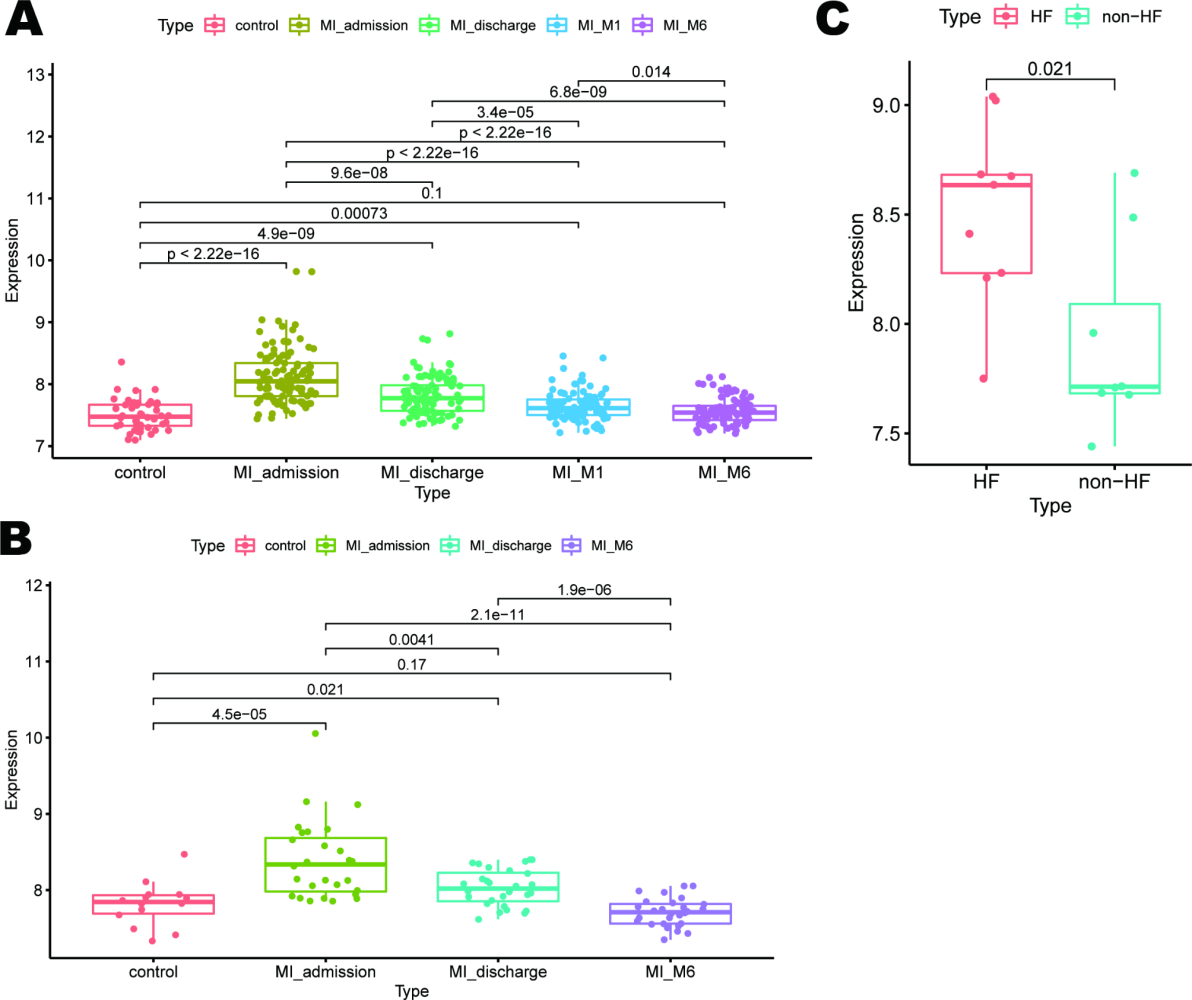
Expression levels during myocardial infarction recovery. (**A**) Expression levels of ADM in the GSE59867 cohort. (**B**) Expression levels of ADM in GSE62646 cohort. (**C**) Expression levels of ADM between the non-HF group and HF group in the GSE59867 cohort.

Finally, we investigated the expression changes of ADM at different times after myocardial infarction. In the GSE59867 cohort, peripheral blood samples at four time points (admission (1st day, n = 111), discharge (4–6 days, n = 101), 1 month after MI (30 days, n = 95), and 6 months after MI (180 days, n = 83) were collected (Fig. 7A). On the first day of MI, expression levels of ADM were significantly elevated relative to control samples (P < 0.001). Over time, the expression of ADM gradually decreased. The ADM expression level in STEMI patients continued to be elevated compared to the control group one month after AMI (P = 0.00073). Six months after AMI, the expression level of ADM returned to the same level as the control group (P = 0.1).

The samples in the GSE62646 cohort have three time points: admission (1st day, n = 28), discharge (4–6 days, n = 28), and 6 months after MI (180 days, n = 28) (Fig. 7B). Consistent with the results from the GSE59867 cohort, ADM expression significantly increased on the first day of MI (P < 0.001), then gradually declined. At 6 months, it returned to control levels (P = 0.17).

Furthermore, in the GSE59867 dataset, patients were divided into HF (heart failure, n = 9) and non-HF group (n = 8) based on NT-proBNP level and LVEF (left ventricular ejection fractions) at 6 months post-MI. To examine the relationship between ADM levels and heart failure after myocardial infarction, we analyzed the expression levels of ADM on the first day of myocardial infarction between two groups. Compared to the non-HF group, the expression level of ADM in the HF group was significantly higher (P = 0.021) (Fig. 7C).

## Discussion

In this study, we first merged datasets from the same platform to minimize possible batch effects and identified 146 DEGs in the integrated dataset. Immune infiltration analysis indicated that eleven cell types were differentially infiltrated, suggesting their potential pathophysiological roles in STEMI. In comparison to the control group, the STEMI samples had increased infiltration of Tregs, monocytes, and neutrophils. Through correlation analysis, we further screened 25 DEGs that showed a high correlation with monocytes and neutrophils. Five genes consistently selected by all three machine learning algorithms were considered candidate genes. We then validated the five candidate genes in terms of their expression and diagnostic efficacy in two external datasets to further identify the candidate genes. ADM had more than 80% high accuracy in all datasets. At last, we evaluated the expression levels of ADM during myocardial infarction recovery and the differential expression of ADM in the heart failure group and non-heart failure group.

The immune system plays a vital role in pre-infarction atherosclerosis, the acute phase of infarction, and the later phase of myocardial remodeling [8, 9]. Functional enrichment analysis of DEGs between the STEMI and control groups was mainly enriched in several immune processes and immune cell-related pathways. Previous studies have demonstrated that multiple cell types are involved in different stages of healing in myocardial infarction, including neutrophils and macrophages [10]. Various inflammatory signals and cell debris massively recruit neutrophils within the first few hours following ischemia [10]. Consequently, monocyte-derived macrophages are recruited to infarcted myocardium to phagocytose cell debris and apoptotic neutrophils [11].

Treg cells are essential for inducing and maintaining immune homeostasis and tolerance, and any disruption in the generation or function of these cells can trigger aberrant immune responses and pathological conditions [12]. Treg cells were mobilized to the infarcted mouse myocardium, regulating fibroblast phenotype and function via its anti-inflammatory properties during the early stage after myocardial injury [13, 14]. A study using a rat model of MI demonstrated that a rise in Treg cell numbers prevented ventricular remodeling and improved cardiac function following MI, through its anti-inflammatory effects and direct protection of heart muscle cells [15]. Weirather et al. reported that therapeutic activation of Treg cells resulted in M2-like macrophage differentiation in the healing heart tissue, accompanied by myofibroblast activation and increased expression of monocyte/macrophage-derived proteins that promoted wound healing [16].

Our study found elevated expression of Tregs, monocytes, and neutrophils in STEMI samples, which is in line with previous research. As there were relatively few genes linked to Tregs in our hub gene screening, we selected differentially expressed genes associated with both monocytes and neutrophils. ADM was positively correlated with monocytes and neutrophils, with correlation coefficients of 0.575 and 0.557, respectively.

ADM, also known as adrenomedullin, is expressed in a variety of tissues, including the vascular system and the heart [17]. Because of its small size (6 kDa), it can easily diffuse between the blood and interstitial fluid [18]. The mature and biologically active hormone ADM is produced through proteolytic cleavage of the full-length precursor protein ProADM [19]. ADM has potent protective functions in various pathological conditions as an endogenous peptide, owing to its anti-oxidant, anti-inflammatory, anti-apoptotic, and proliferative properties [20, 21]. Trincot et al. reported that [22] adrenomedullin drives reparative cardiac lymphangiogenesis and function via Cx43 to preserve cardiac function and reduce edema. It has been reported that elevated expression of ADM is found in cardiomyocytes exposed to simulated ischemia, suggesting paracrine effects that could decrease cardiomyocyte apoptosis [19]. A study report reveals that Bio-ADM serves as a useful predictor and biomarker of impaired hemodynamics in cardiogenic shock patients. Elevated levels of Bio-ADM may indicate the development of refractory shock and organ dysfunction [23]. In our study, the expression levels of ADM significantly increased during an acute myocardial infarction and decreased gradually over time. The AUCs of ADM for all datasets were greater than 0.8, exhibiting the strong diagnostic power of ADM. In addition, we analyzed the expression levels of ADM on the first day of myocardial infarction between the non-HF and HF groups. The HF group exhibited a significantly higher level of ADM expression than the non-HF group, which suggested that patients with high levels of ADM expression during a heart attack were more likely to develop heart failure.

Our study also has some limitations. This research is a bioinformatics analysis. The specific mechanisms by which ADM interacts with monocytes and neutrophils during STEMI, as well as the downstream effects of this interaction on myocardial damage and long-term prognosis should be further explored. Additionally, investigations into the potential therapeutic benefits of targeting ADM or its downstream effects could open up new avenues for improving the clinical outcomes of STEMI patients.

## Conclusion

In this study, we identified a candidate diagnostic marker (ADM) in STEMI peripheral blood from an immune molecular perspective, which might provide a potentially new mechanism of STEMI. ADM positively correlated with monocytes and neutrophils, suggesting its potential role in the immune response during STEMI. Moreover, ADM was confirmed as an excellent diagnostic marker by expression level and diagnostic efficacy in another two external datasets. The findings might help to develop new diagnostic tools or therapeutic strategies for STEMI.

## Electronic supplementary material

Below is the link to the electronic supplementary material.

Supplementary Material 1

Supplementary Material 2

Supplementary Material 3

Supplementary Material 4

Supplementary Material 5

## Data Availability

The datasets presented in this study can be found in the GEO database: https://www.ncbi.nlm.nih.gov/geo/.
