# Supplementary material for: Exploring an immune cells-related molecule in STEMI by bioinformatics analysis
Source: BMC Med Genomics. 2023 Jun 30;16:151. doi: 10.1186/s12920-023-01579-8 (PMC10311814; doi:10.1186/s12920-023-01579-8)
Supplement: Supplementary file 1 — Supplementary Material 1 [file 12920_2023_1579_MOESM1_ESM.pdf]

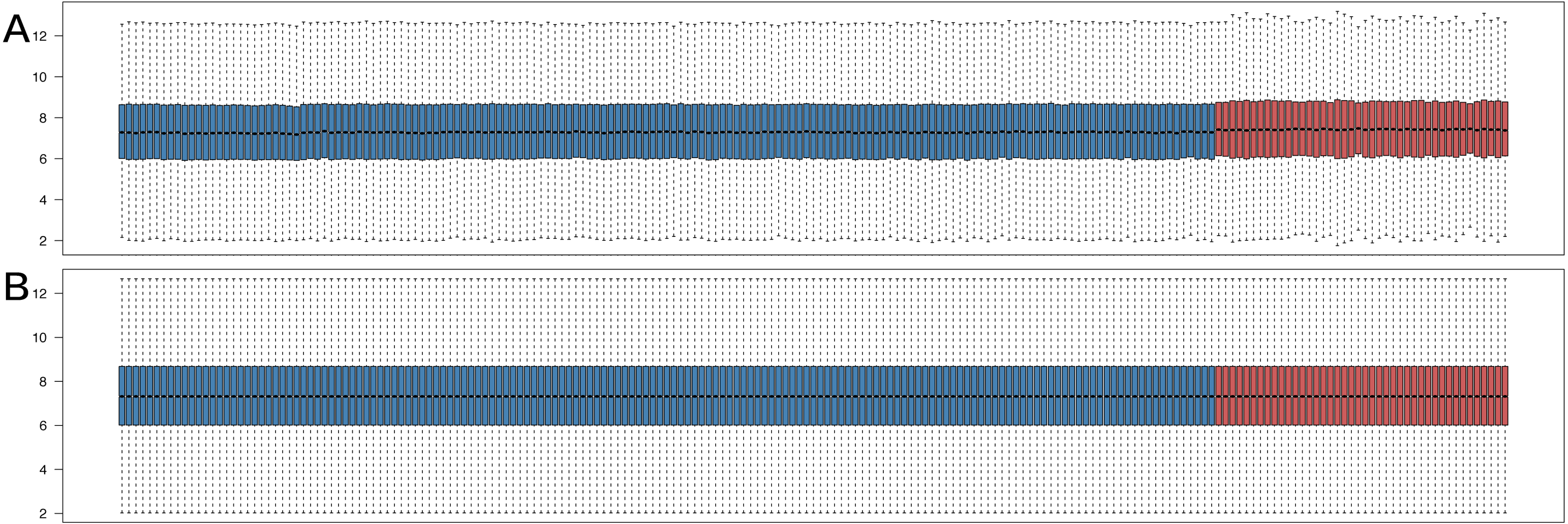

Figure S1 Data preprocessing. Boxplots were performed to remove batch correction of the metadata cohort. (A) before batch correction and (B) after batch correction. Blue and red boxplots represented GSE59867 and GSE62646 cohorts, respectively.
