# Supplementary figures and images for: Exploring an immune cells-related molecule in STEMI by bioinformatics analysis
Source: BMC Med Genomics. 2023 Jun 30;16:151. doi: 10.1186/s12920-023-01579-8 (PMC10311814; doi:10.1186/s12920-023-01579-8)

## nomoscore

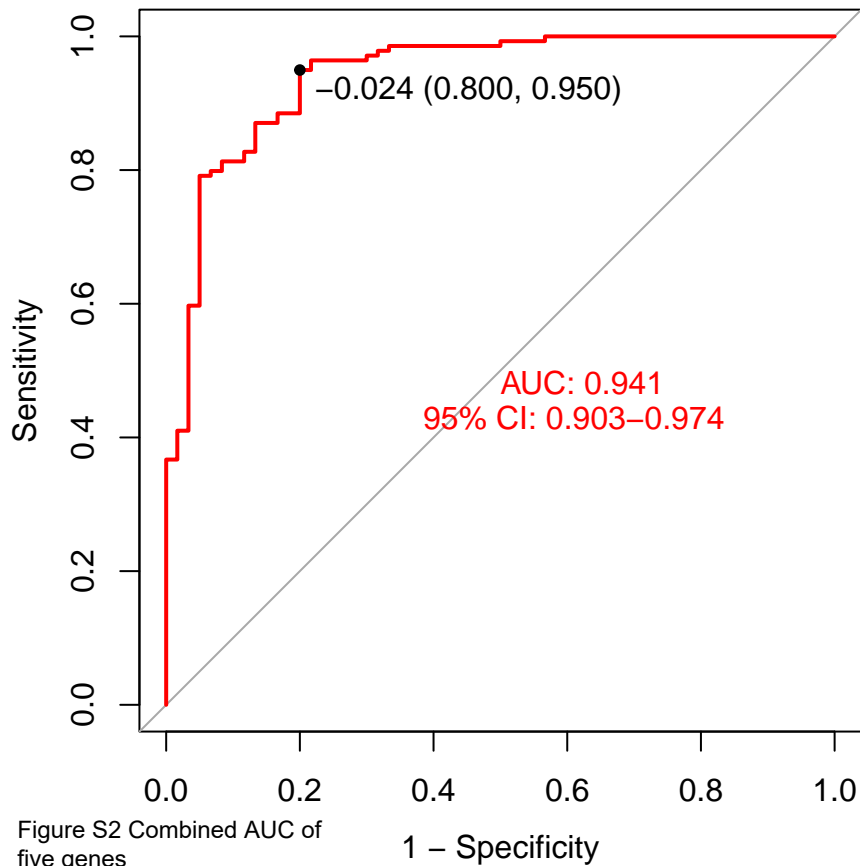

Figure S2 Combined AUC of five genes

Supplement: Supplementary file 2 — Supplementary Material 2 [file 12920_2023_1579_MOESM2_ESM.pdf]

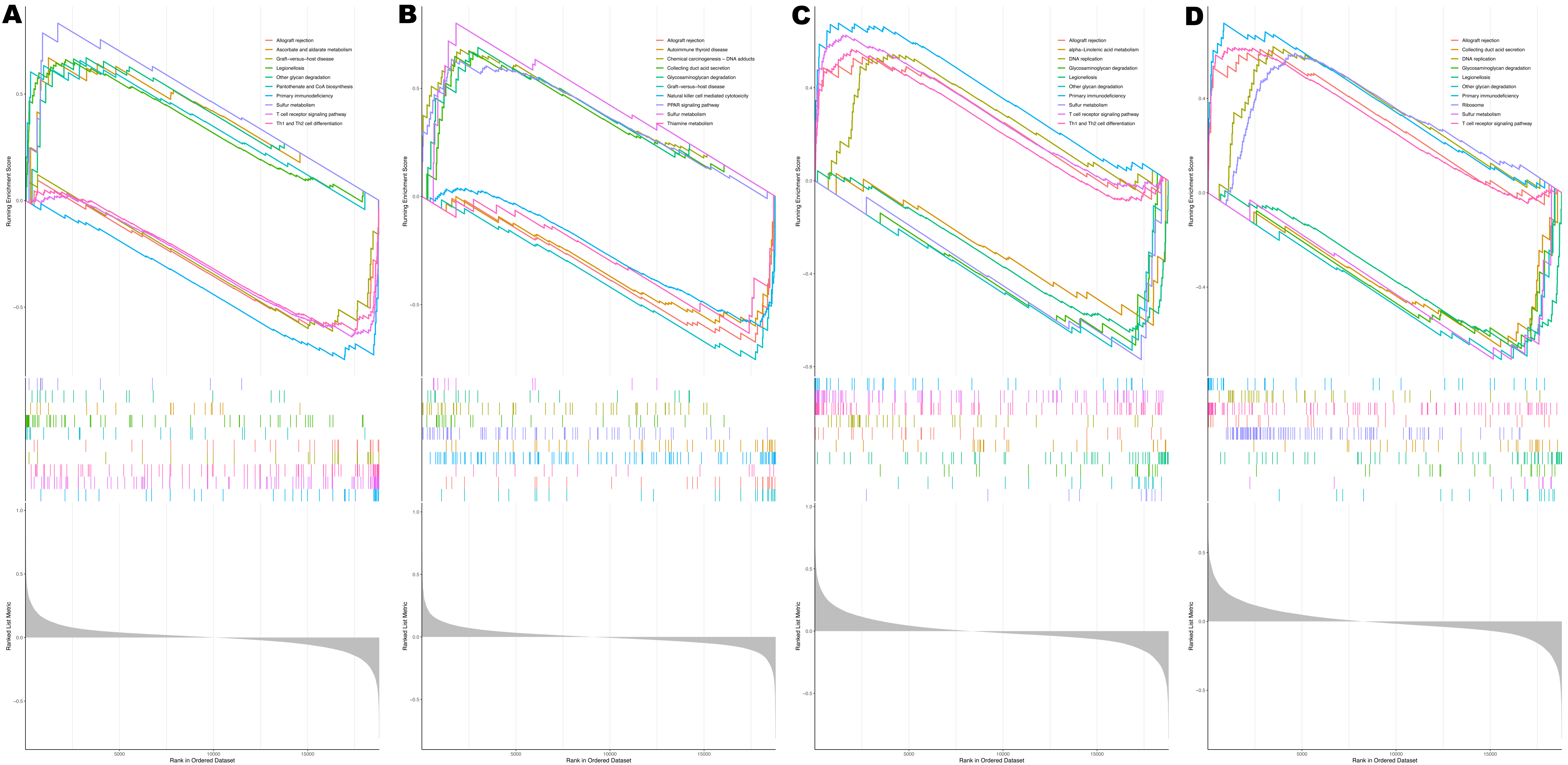

Figure S4 Gene set enrichment analysis of the other four hub genes.

Supplement: Supplementary file 4 — Supplementary Material 4 [file 12920_2023_1579_MOESM4_ESM.pdf]
