# Supplementary material for: Exploring an immune cells-related molecule in STEMI by bioinformatics analysis
Source: BMC Med Genomics. 2023 Jun 30;16:151. doi: 10.1186/s12920-023-01579-8 (PMC10311814; doi:10.1186/s12920-023-01579-8)
Supplement: Supplementary file 3 — Supplementary Material 3 [file 12920_2023_1579_MOESM3_ESM.pdf]

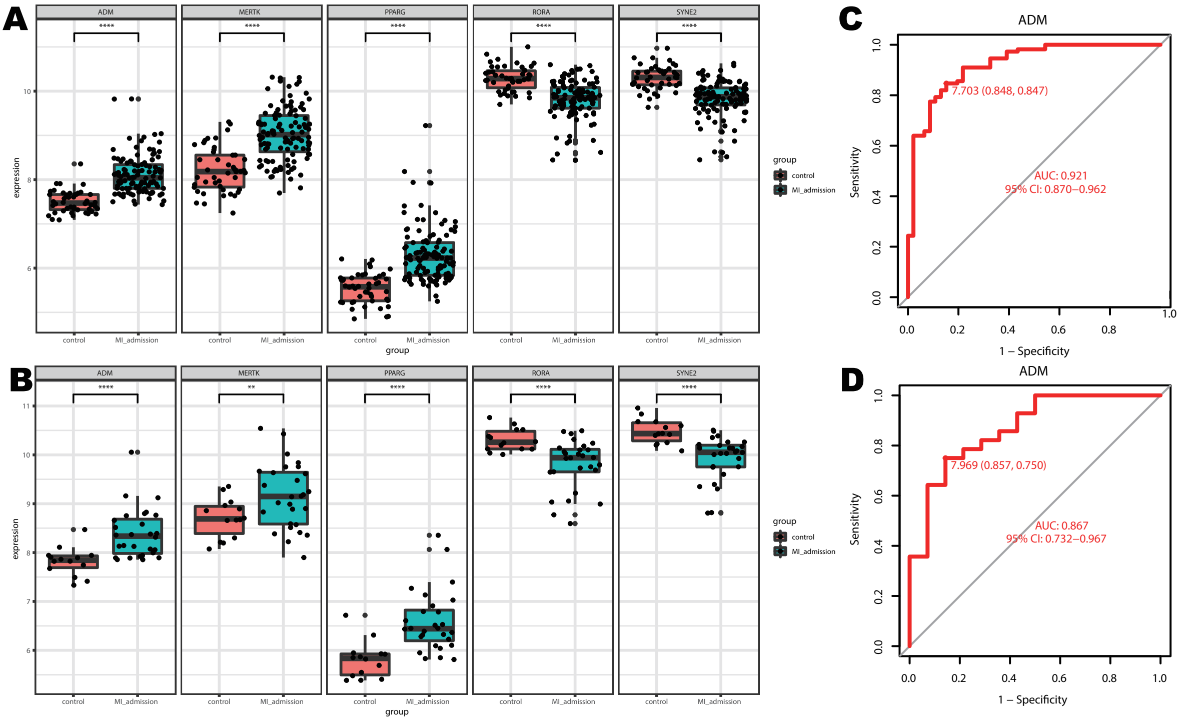

Figure S3 Validation of candidate diagnostic markers. (A) Expression of hub genes in GSE59867 cohort. (B) Expression of hub genes in GSE62646 cohort. (C) ROC curves of ADM in the GSE59867 cohort. (D) ROC curves of ADM in the GSE62646 cohort.
